# From Data to Improvement: Social Mechanisms as a Key to Understanding Dashboard Adoption

**DOI:** 10.1097/JMQ.0000000000000225

**Published:** 2025-02-06

**Authors:** Tamara Broughton, Anne Marie Weggelaar-Jansen, Sandra Sülz

**Affiliations:** 1 Tranzo, Tilburg University. Tilburg, The Netherlands; 2 Erasmus School of Health Policy & Management, Erasmus University, Rotterdam, The Netherlands

**Keywords:** quality dashboard, quality improvement, qualitative research, social mechanisms

## Abstract

Research on dashboard adoption has focused on technical and design requirements. Evidence on social mechanisms for successful dashboard adoption is scarce. This study examined 2 quality dashboards in a similar organizational context with different outcomes. The research question was: How do social mechanisms influence the adoption of dashboards in practice? This embedded case study within one Dutch hospital in 2 phases: (1) interviews and observations to identify social mechanisms in the end-user’s team and (2) expert focus groups to validate identified mechanisms. Data were transcribed verbatim and analyzed thematically, resulting in the identification of 3 social mechanisms within the team of end-users influencing dashboard adoption: cultivating a supportive team climate, trust, and leadership behavior in end-users’ teams. These mechanisms stimulate a learning environment for discussing and improving care quality. They require action from individuals and teams, so dashboards can be used for collective understanding, learning, and improving. Without these social mechanisms, dashboards remain an unadopted “materiality.”

## Introduction

In health care, increasing numbers of dashboards have been developed to provide a visual overview of various indicators to either aid health care professionals in clinical decision-making or inform management at a unit, ward, or organizational level.^1^ The growing interest in value-based health care (VBHC)^2^ has spurred the growth of (patient-reported) outcome-focused dashboards. Evidence suggests that implementing and adopting dashboards can result in enhanced patient outcomes, especially due to improvements in communication and information sharing (eg, Koopman et al^3^ and Daley et al^4^). With dashboards having the potential to contribute to improvement work, it is important to understand the mechanisms contributing to successful dashboard adoption to leverage this potential. Therefore, this team studies the adoption process of 2 dashboards, aiming to improve care quality, within one hospital to identify these mechanisms.

Research reveals significant dashboard design variation^5^ and emphasizes the importance of human-centered, interdisciplinary design^6^ and prototyping based on technical requirements.^7^ In addition, dashboard visualization requires data quality (eg, timely, complete), correct data display (eg, color-coding), and content flexibility for specific end-users.^1,8,9^ Equally important to the design and technical requirements is the actual adoption of the dashboard. Previous studies have identified several challenges associated with dashboard adoption, including lack of rigorous data sources, identifying meaningful content, unclear design, and lack of integration with other hospital systems.^10^ Furthermore, Reszel et al^11^ show how the same dashboard triggered variable effects on care improvement across different hospitals. This evidence illustrates that dashboards are not magic bullets that automatically improving the care quality. Instead, dashboard adoption is also shaped and influenced by socio-organizational factors,^12^ such as integration in quality improvement behavior and enhancing the dashboards’ flexibility and connectivity to serve as an improvement tool. This work contributes to this literature stream by studying dashboards’ adoption processes aimed at improving care quality and safety. Many theories, models, and frameworks use (slightly) different definitions for technology adoption in health care (see the studies by Rad et al^13^ and Heinsch et al^14^ for an overview), sometimes using adoption, diffusion, and implementation interchangeably. This study defines adoption as the actual use of dashboards in practice, with attention for the interaction between the innovation, intended adopters, and context.^15^ Dashboard adoption extends design and technical requirements, encompassing also individual factors and social mechanisms such as human interaction and embedding into daily routines and mundane practices.^15^ Therefore, this study is especially interested in the dynamics of social mechanisms during dashboard adoption processes within the team of end-users. The research question guiding this study is as follows: How do social mechanisms influence the adoption of dashboards in practice?

## Methods

This research team conducted an embedded case study^16^ and reported in line with the COREQ-checklist.^17^ The context was a teaching hospital in the Netherlands with 425 inpatient beds, 252 specialized physicians, and a total of 3265 employees. Two disease-specific dashboards served as units of analyses in a similar organizational context.^16^ The dashboards were developed at the same time, in the same hospital, by the same dashboard developers, with the same aim (eg, stimulating internal learning purposes to enhance quality and safety). Only the end-users differed between the 2 adoption processes, helping us to understand how the dashboards were adopted and why sometimes adoption was more successful.

A qualitative design was used in 2 phases. In phase 1, the first author conducted 11 semistructured interviews and 4.5 hours of participatory observations over a 13-month period to determine important themes related to dashboard adoption. For the semistructured interviews, respondents were purposively selected by the first author who worked at the hospital but was not actively involved in the development of these dashboards. Respondents were chosen based on their involvement in the development and adoption of the studied dashboards, that is, end-users [physicians and nurses (N = 3, of which 2 males and 1 female)], support staff [data analysts and quality advisors (N = 5, of which 1 male and 4 females)], and managers (N = 3, of which 1 male and 2 females). For the interviews, respondents were invited either by email or face-to-face with a written explanation of the study. All selected respondents agreed to participate. In addition, 3 quality improvement meetings using the dashboard were observed to capture the interactions of actors. Combining interviews with participatory observations allowed for triangulation of different data sources.

Semistructured interviews focused on the chronology of the dashboard development and implementation. In addition, predefined topics were discussed, such as the development process, team involvement in the development, process successes, improvements for the future, and the current adoption (topic list provided in Supplementary Material 1, Supplemental Digital Content 1, available at http://links.lww.com/AJMQ/A134). Interviews lasted 52 minutes on average and were conducted face-to-face or via Microsoft Teams, based on respondents’ preference. Audio (face-to-face) or video (Microsoft Teams) recorded interviews were transcribed verbatim. Thick descriptions (ie, detailed, contextualized account of social actions and interactions capturing not only the behavior but also the underlying meanings, intentions, and cultural context) were made of the observed quality improvement meetings.^18^ Using ATLAS.ti (v. 23), a thematic analysis as described by Braun and Clarke^19^ was performed on the collected data, and 3 social mechanisms were identified in an inductive way. Data saturation was achieved after the interviews and observations.

In phase 2, the first author presented the identified social mechanisms during 2 digital focus groups with VBHC experts (N = 35; for group composition, see Supplementary Material 2, Supplemental Digital Content 1, available at http://links.lww.com/AJMQ/A134). The 2 focus groups lasted 1 hour, were held online via Microsoft Teams, and were processed in a similar manner as the interviews. The social mechanisms were presented during the focus groups for validation and generalization and to deepen the researchers’ understanding.

The Ethics Review Board of Tilburg University (Reference: TSB_RP1196) provided ethical approval. Data storage is compliant with the General Data Protection Regulation as pseudonymization of data followed transcription. All participants provided consent.

## Results

The research team studied the 2 dashboard adoption processes aimed to stimulate internal learning to improve quality and safety. Both studied dashboards present medical outcomes, patient-reported outcomes, and process outcomes of a specific patient group, in line with the VBHC principles. Despite sharing the same context, a different adoption outcome was observed. One was successfully adopted. It is used approximately 4 times per year during regional quality improvement meetings with physicians and nurses, whichcontributes to ongoing improvement activities. The other dashboard did not result in any quality improvement activities, nor was discussed in meetings. The nonadoption became especially evident when the dashboard needed an update to incorporate a new dataset, but this update was never requested nor done. The findings indicate that (non)adoption of dashboards is influenced by 3 social mechanisms: cultivating a supportive team climate, ensuring trust, and displaying leadership behavior within the team. In the results, we will show that social mechanisms are not constrained to formal meetings, but also appear in the form of meeting culture and patterns of social interaction.

### Cultivating a Supportive Team Climate

The analysis of the data showed that different aspects of team climate influenced dashboard adoption in daily practice. First, a friendly atmosphere among colleagues (eg, joking, shaking hands or a slap on the shoulder at the start, spontaneous private conversations) contributed to using the dashboard as input for an open and honest conversation about the care quality. Feeling safe within the team was equally important, especially when the dashboard contained concerning information for the end-users (eg, high complication rates). If at least one team member felt safe enough to address the confronting information and potential mistakes made, this opened-up the conversation about the daily practice behind the indicators in the dashboard and enabled discussing options to improve quality.

Furthermore, it was noticed how, while discussing complications during the meeting, the team members used respectful language to prevent reputational damage when addressing colleagues responsible for the complication. For example, they would start their improvement suggestion with: “It is easy to say this when knowing that a complication will occur, but what I would have done …” or “Of course I wasn’t there, but…” (Observation report quality improvement meeting 3). This respectful communication style contributed to a safe atmosphere, which in return positively contributed to a safe environment for team members to discuss different practices and thereby learn from each other. Dealing with confronting information in a respectful way circumvented the perception that the dashboard could be used for shaming and blaming individuals or the entire team, creating a supportive team climate for learning. This is underlined by an extract from a quality improvement meeting:

“She always discusses the data in such a pleasant and respectful way, so I don’t have the feeling we are talking about mistakes or faults” (Observation report quality improvement meeting 3)

Finally, the quality improvement meetings of one team were always combined with an informal dinner. During dinner, there was room for reflections on previously discussed topics and casual conversation, positively contributing to the team climate. Combining the quality improvement meetings with social activities also made them more attractive and ensured high attendance. This contributed to (social) interactions with and about the dashboards thereby positively influencing the adoption process.

### Ensuring Trust

The importance of ensuring trust was observed in different forms throughout this study. First, it was important that the team trusted the concept of using health care outcome data, visualized in a dashboard, as part of their quality improvement strategy. If dashboards were seen as serving the purpose of facilitating learning, it became self-evident that measuring health care outcomes was part of the quality improvement cycle:

“Measuring is knowing, you want to know how you’re doing, and then you want to do better.” (R6)

But dashboards could also be seen as serving the purpose of external benchmarking, a purpose that was partially feared, questioned, or mistrusted:

“There are insurance company initiatives in which these kind of things [red: outcome indicators] were often put on the agenda. What we noticed was that independent treatment centers, specialized in this particular treatment, were seen as the benchmark, and you had to stay within a few percent from them. And that’s when I thought: hold on, we are comparing apples and pears here […] Eventually I noticed that [name insurance company] went for a run with these kinds of numbers and saw it as quality improvement. And I don’t know whether that’s the case.” (R7)

The respondent found this benchmarking method unfair because the patient mix of independent treatment centers is not comparable with hospitals since independent treatment centers only operate low-risk patients with standardized routine treatment procedures. During the interview, s/he also expressed that s/he did not trust that the indicators on the dashboard would only be used for quality improvement. Rather s/he suspected that indicators on the dashboards were also used for regulatory purposes either internally or by insurance companies, undermining the respondents’ trust. This mistrust hampered the adoption of the dashboard and illustrates the importance of end-users fully trusting the dashboard as input for their quality improvement work only.

In addition, the importance of trusting the data was observed multiple times. When the team did not believe that the dashboard could be used as part of their quality improvement strategy, this was normally voiced through discussions about (mis)trusting the data. During the observed meetings, much time was spent on discussing whether the displayed data were correct. When participants did not believe that the dashboard was part of their quality improvement strategy, these discussions were longer, and it was difficult to move the conversations from data accuracy to data as input for the conversation about quality improvement. One respondent, whose dashboard was eventually not used in quality improvement work, expressed this as follows:

“I have always said: I am skeptical about what we are measuring, because it is very easily influenced by how you address it. And I always had the feeling that there are so many factors that influenced the indicators, so it is questionable whether you can associate it with what a doctor has done or changed.” (R7)

Finally, the data showed how dashboards could also facilitate trust and collaboration among team members. Due to the introduction of volume norms that hospitals need to meet to perform certain procedures, physicians from different hospitals had collaborated to meet the threshold for certain norms. The physicians used the dashboard to facilitate their collaboration and to gain trust in each other skills, as illustrated by the following quote:

“Different people were going to operate here, in our ‘toko’ [red: slang word for our place]. So, we wanted to prevent accidents from happening. We knew who we were letting in, let me be clear, but we also wanted to compare [red: our outcomes] with each other and in the beginning, we also looked at each other’s operations in the OR and the results in the dashboard. Later on, we knew: it’s alright.” (R6)

This excerpt shows how the dashboard was used to gain trust in each other’s work, by checking and monitoring each other’s outcomes, using them for a proper discussion on how procedures were performed. Once trust had been established, dashboards no longer fulfilled a monitoring purpose but rather facilitated improvement discussions. This not only illustrates how the dashboard itself improved trust and collaboration between physicians of different hospitals but also indicates that the role and purpose of dashboards can change for the same users.

### Displaying Leadership Behavior

Displaying leadership behavior played another crucial role in the adoption process. In this study, each team had a medical chair during the dashboards’ development. Medical chairs expressing a clear vision of the dashboard content and recognizing its value facilitated the dashboard adoption:

“The nicest thing about it [red: implementation of the patient reported outcomes measures (PROMs) for the dashboard] was that the physician [red: medical chair] really saw the added value of the PROMs. […] He thought it was fantastic that we were going to measure this, because in his/her opinion this were the outcomes that really mattered.” (R1)

It was also observed that chairs who explicitly articulated that they thought the dashboard could contribute to quality improvement and clearly communicate this to the team, positively contributed to the dashboards’ adoption. As described by one of the data analysts involved in the adoption process:

“I think that the most important thing [red: throughout the development] was that he [red: the medical chair] was a good ambassador of the dashboards, because if you don’t have someone that promotes it, then it won’t land, and nothing will happen with it.” (R3)

In addition, it was found that the previously described safe atmosphere and team climate was facilitated by the way quality improvement meetings were chaired. For example, in one meeting, complications of procedures were analyzed using videos edited to conceal the identity of the colleague performing the procedure. The medical chair stated explicitly that the video served learning purposes, which did not require revealing the colleague’s identity. This created a climate to thoroughly discuss the procedure and associated complication. Ultimately, the colleague in question even felt safe enough to disclose that s/he performed the procedure, which contributed to better understanding circumstances and enhanced the overall learning experience.

Notably, displaying leadership behavior to facilitate learning was not confined to medical chairs. For example, in one quality improvement meeting, a discussion about data accuracy of the complication indicator was avoided by a physician arguing that every complication is unfortunate for a patient, no matter what caused it. The physician showed personal leadership and called for an open discussion about possible causes for the increased complications such that improvement strategies could be identified.

## Discussion

This study aimed to enhance the understanding of social mechanisms influencing dashboard adoption in health care practice. Successful adoption is realized when dashboards are used for learning to improve the care quality. Thus, this includes the adoption of both the dashboards’ “materiality” (technical) (eg, Khairat et al^7^ and Ghazisaeidi et al^20^) and a learning process based on the displayed information on the dashboard (social).^12^ The findings align with previous research demonstrating that the “materiality” does not automatically lead to quality improvement (eg, Dowding et al^1^). The dashboards studied shared the same organizational context, and similar technical and design elements; yet the adoption outcomes differed. This study shows different interlinked aspects influencing the adoption. Several health care technology adoption frameworks emphasize that different domains are inextricably interlinked and dynamically evolving, making adoption in practice complex.^14,21^

To understand the differences between the studied adoption processes, it is important to realize that dashboards in health care can have different purposes for different users. First, health care professionals use dashboards to aid decision-making for patients and stimulate internal learning purposes for quality improvement (eg, Daley et al,^4^ Reszel et al,^11^ and Stone et al^22^). Second, managers use dashboards to gain insights about progress and performance at the department or organization level.^12,23^ Finally, external parties, such as insurance or inspectorate bodies, use dashboards for monitoring, benchmarking, and accountability (eg, Overveld et al^24^). Although one could argue that different purposes necessitate different dashboard types and/or indicators, it must be acknowledged that different purposes can coexist, consequently influencing user adoption. Previous work highlighted a tension between using dashboards internally for learning and externally for monitoring and accountability.^25^ Especially in quality improvement, a dashboard should stimulate discussion, while for other purposes, dashboards are used to monitor and direct.^26^ The empirical evidence illustrates this tension, since some respondents feared their quality improvement dashboard would be used for benchmarking purposes by external parties, which hampered the adoption of the dashboard for quality improvement work. This tension between purposes asks for more than previously studied technical and organizational requirements to successfully adopt dashboards for quality improvement. The results show that social mechanisms play an important part here.

This study illustrated how a safe team climate was created by using respectful language when discussing the dashboard indicators and possible errors made by physicians. Also, how a friendly atmosphere was created and nurtured during quality meetings by using humor and taking time for social conversations. This created an atmosphere in which the dashboard could be used as input for an open and honest conversation, stimulating the discussion needed for learning purposes. This study found that social mechanisms creating trust within the team influenced how the dashboards were used for learning. Without trust, teams spent most of their meeting discussing the accuracy of the displayed data, avoiding discussions about what they can learn from the data and related quality improvement strategies. Conversely, when trust was observed, the team focused their discussions on potential ways to improve patient outcomes, facilitated by learning from the data.

Finally, this study revealed how different team members, not limited to formal leadership (ie, the medical chair of the meetings in our study), played an important part in facilitating social processes for adoption. The importance of individuals in the successful adoption has previously been discussed in champion literature.^27,28^ Most research report the presence or absence of champions in implementation studies (eg, Poba-Nzaou et al^29^), whereas the empirical data shows how a champion can contribute to a psychological safe environment.^30^ By refraining from using physicians’ names when discussing complication or actively calling for an open discussion about possible causes behind lower scoring indicators without labeling them as mistakes, an environment in which the team felt safe to voice ideas, to give and receive feedback was created. This enabled the local learning process essential for dashboard adoption.

These empirical findings show that the coexistence of varying purposes, creation of a safe team climate, and champions have an active role in creating a learning environment needed for dashboard adoption. This resonates with recent work by Bransby et al^31^ calling for a multilevel perspective in which individual (champions), climate (safe team climate), and context (varying purposes) factors can influence psychological safety, which enables learning.^31^ In addition, Bransby et al^31^ emphasize that psychological safety is not default in workplaces but must be actively cultivated and nurtured.^31^ This resembles the argument that action on social mechanisms is required to successfully adopt dashboards in practice, either through building a safe team climate, creating trust, or champions contributing to a psychological safe environment.

## Limitations

This study has 3 main limitations. First, the first author, an employee of the studied organization, did not participate in dashboard development but was involved in postdevelopment quality improvement meetings. This position facilitated easy access to respondents and enhanced understanding of dashboard adoption. However, her close association with respondents might have influenced their responses, potentially leading to exaggerated positive contributions or voiced concerns. Therefore, focus groups were essential to validate and complement respondents’ answers.

Second, this study focused on 3 team-level social mechanisms influencing dashboard adoption, despite other potential influences acknowledged by technology adoption models^14,21^ and highlighted in the focus groups, such as the impact of higher managements or broader organizational context. We welcome studies that study the interplay of different levels. While numerous (social) mechanisms could affect adoption; this study prioritized the most prominent social influences observed in teams we studied and were corroborated by the focus groups.

Finally, while the single-organization design allowed for a detailed microlevel analysis of team-level adoption, it limited exploration of different organizational contexts, which might be very relevant for successful dashboard adoption for learning to improve the care quality.

## Implications for Future Research and Practice

To enhance the understanding of dashboard adoption, future research should extend to other health care organizations to investigate the role of the organizational context and reveal additional social mechanisms. In addition, the influence of the macrolevel, meaning national or international programs or policies on data-driven quality improvement such as VBHC, could be considered, as this will evolve the understanding of all the different factors influencing dashboard adoption.

In practice, these findings can help quality improvement teams struggling to achieve quality improvement based on the data displayed on the dashboard. In addition, this study warns management to not assume that dashboards will automatically enhance improvements as many interrelated social mechanisms influence successful dashboard adoption.

## Conclusion

From a social perspective, this study has shown that social mechanisms, such as building a safe team climate, creating trust, and supportive leadership behavior influence the successful adoption of dashboards in practice. Together these mechanisms stimulate a learning environment in which care quality and safety can be discussed and ultimately improved. These mechanisms require individual and team action, such that dashboards can be used in the puzzle of collective understanding, learning, and improving. Without these, active social mechanisms’ dashboard will not be used in quality improvement work, but simply remain a nonadopted “materiality.”

## Conflicts of Interest

The authors have no conflicts of interest to disclose.

## Author Contributions

All authors contributed to the study conception, design and material preparation. Data collection was performed by Broughton. Data analysis was performed by all authors. The first draft of the manuscript was written by Broughton and all authors commented on previous versions of the manuscript. All authors read and approved the final manuscript.
